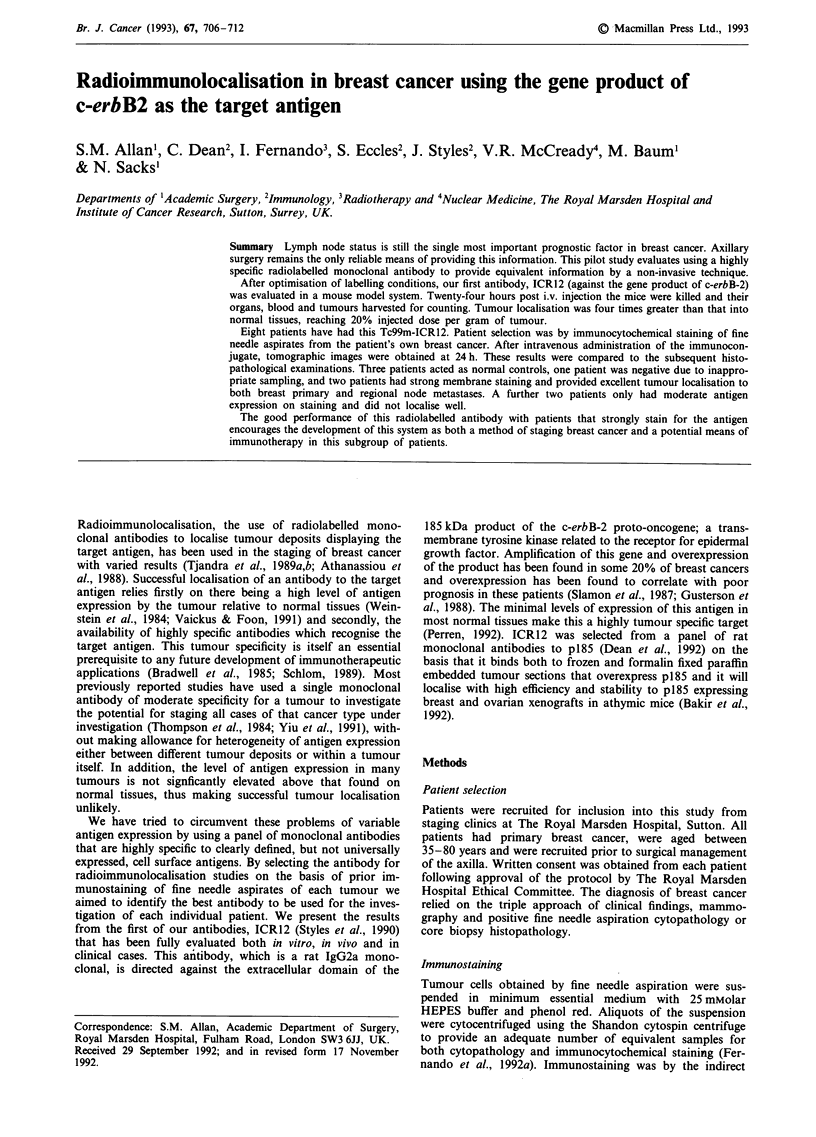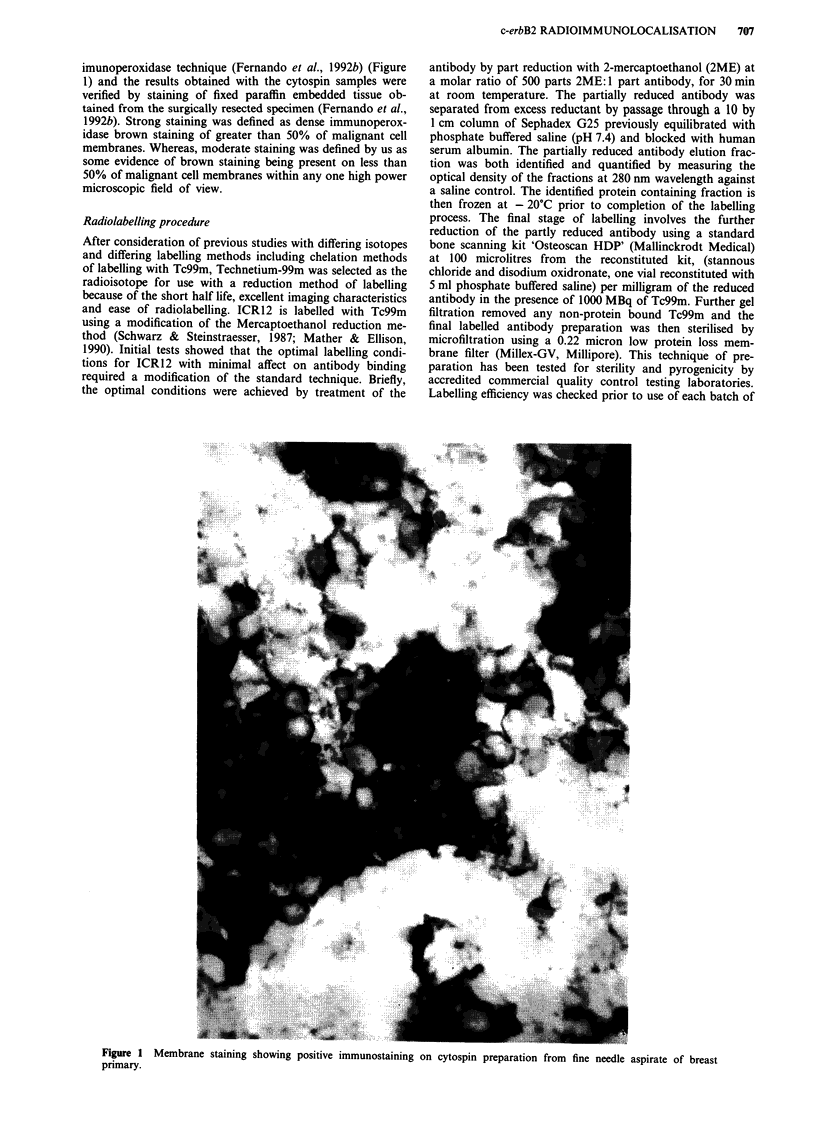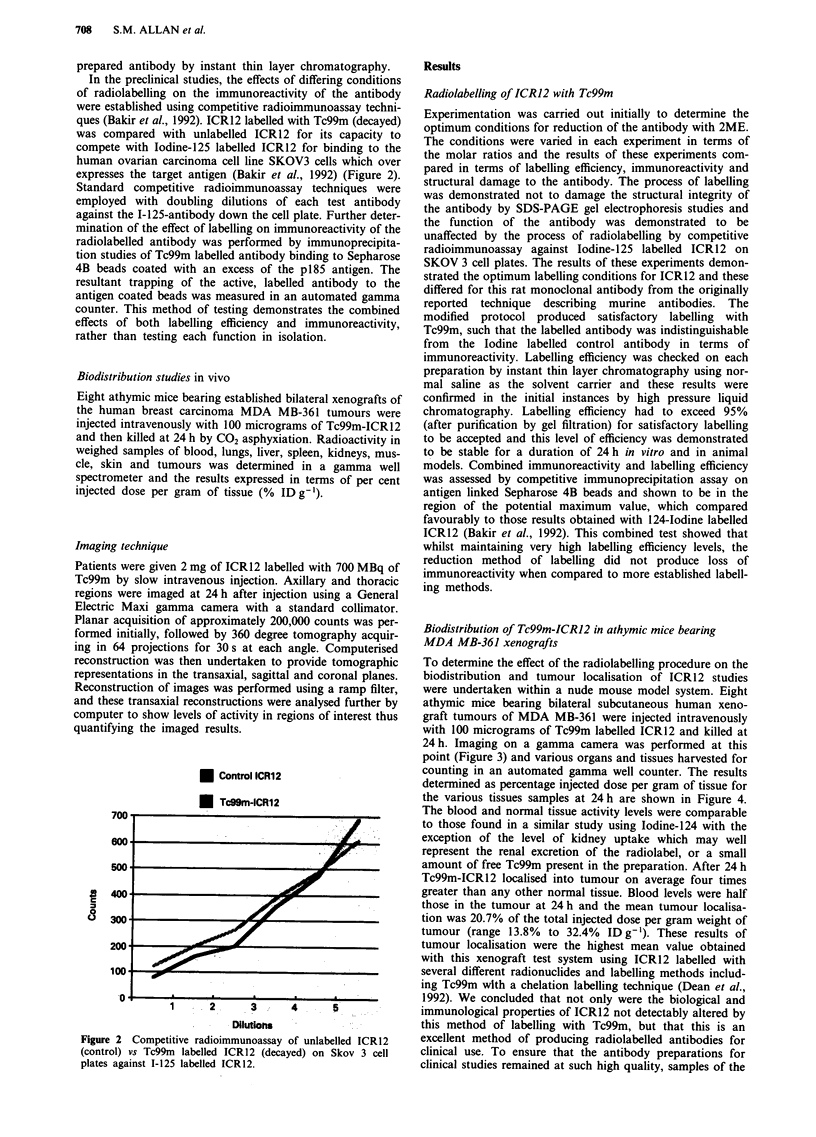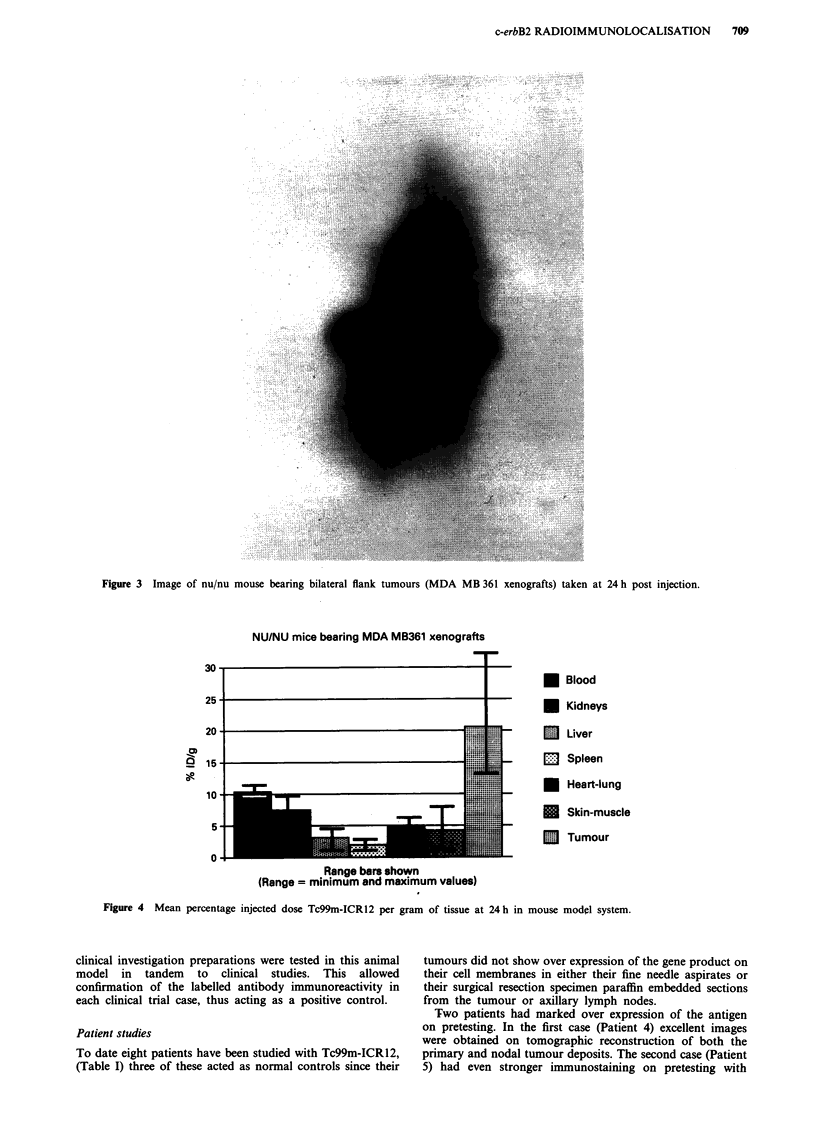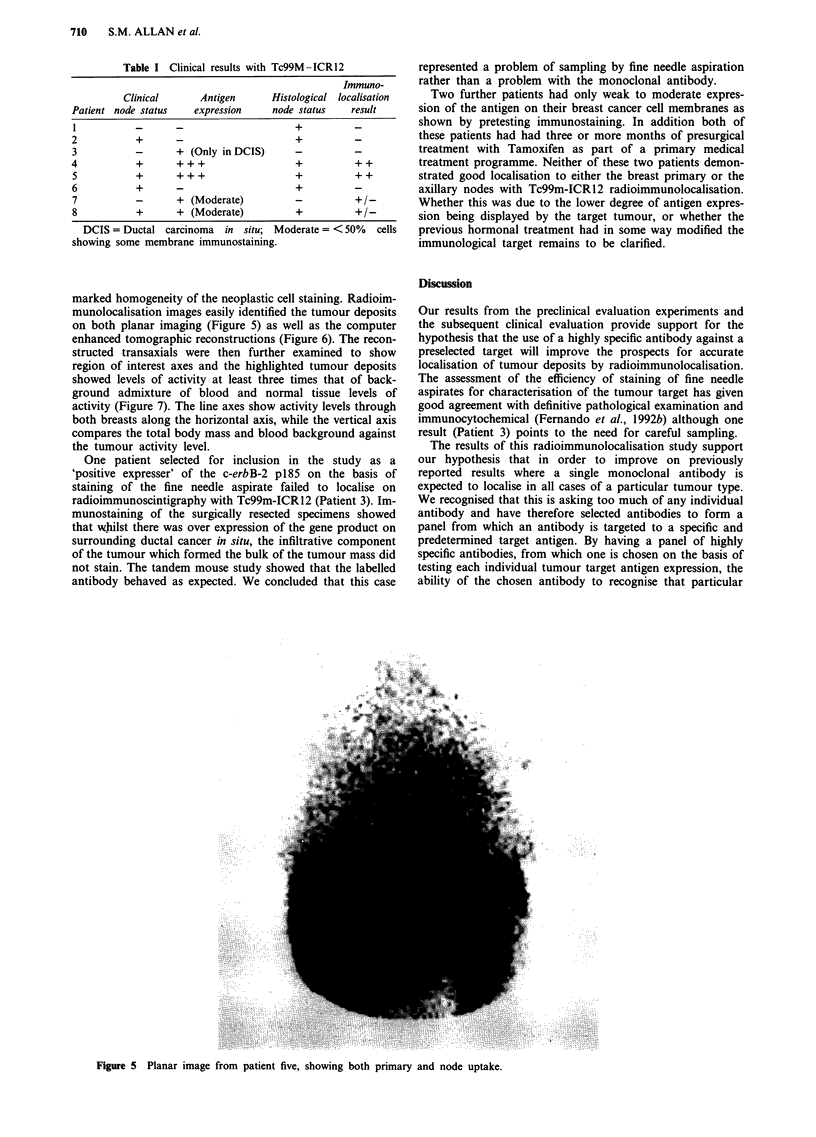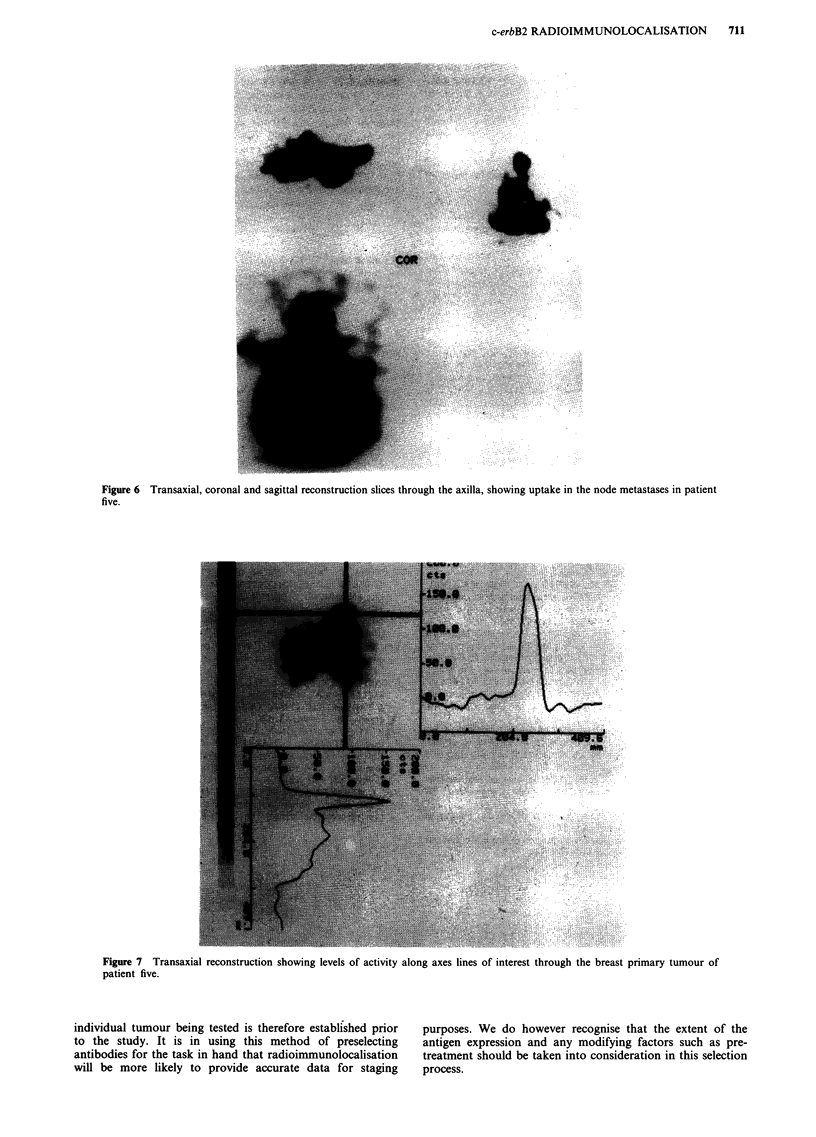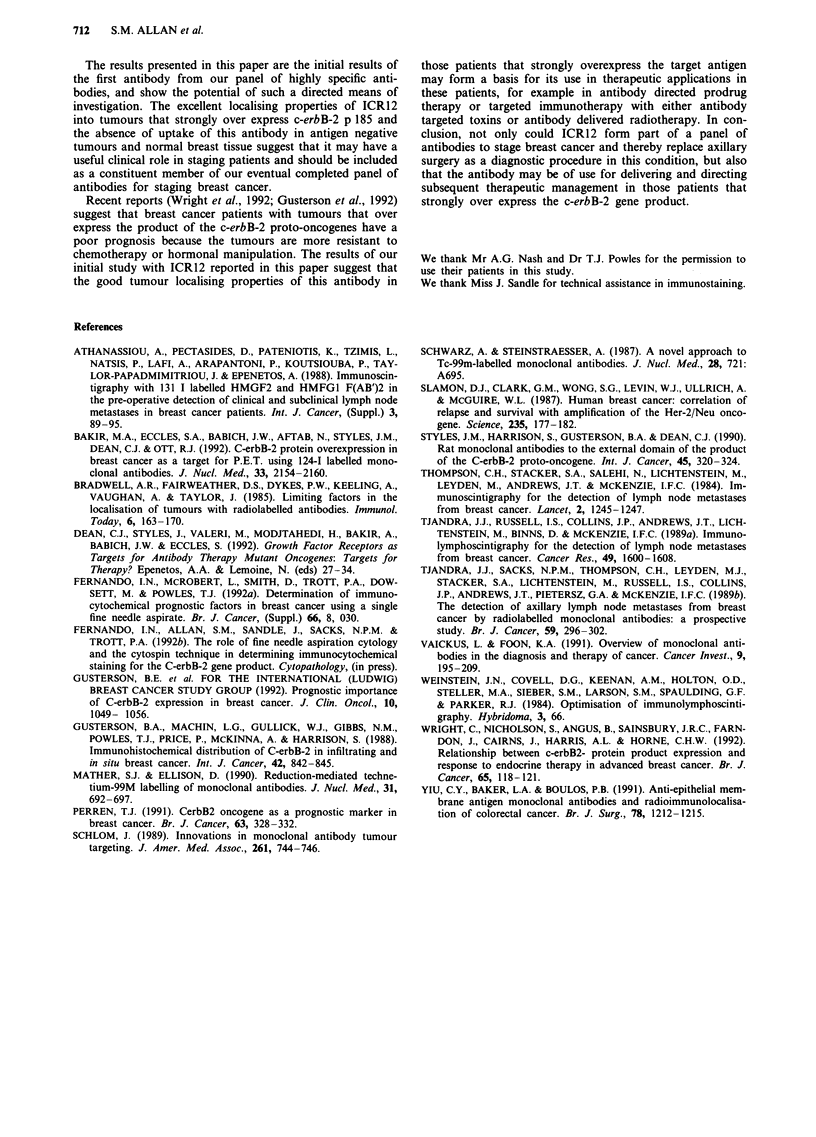# Radioimmunolocalisation in breast cancer using the gene product of c-erbB2 as the target antigen.

**DOI:** 10.1038/bjc.1993.130

**Published:** 1993-04

**Authors:** S. M. Allan, C. Dean, I. Fernando, S. Eccles, J. Styles, V. R. McCready, M. Baum, N. Sacks

**Affiliations:** Department of Academic Surgery, Royal Marsden Hospital, Sutton, Surrey, UK.

## Abstract

**Images:**


Br. J. Cancer (1993), 67, 706–712 © Macmillan Press Ltd., 1993

### Radioimmunolocalisation in breast cancer using the gene product of c-*erb*B2 as the target antigen

S.M. Allan[1], C. Dean[2], I. Fernando[3], S. Eccles[2], J. Styles[2], V.R. McCready[4], M. Baum[1] & N. Sacks[1]

*Departments of [1]Academic Surgery, [2]Immunology, [3]Radiotherapy and [4]Nuclear Medicine, The Royal Marsden Hospital and Institute of Cancer Research, Sutton, Surrey, UK.*

**Summary** Lymph node status is still the single most important prognostic factor in breast cancer. Axillary surgery remains the only reliable means of providing this information. This pilot study evaluates using a highly specific radiolabelled monoclonal antibody to provide equivalent information by a non-invasive technique.

After optimisation of labelling conditions, our first antibody, ICR12 (against the gene product of c-*erb*B-2) was evaluated in a mouse model system. Twenty-four hours post i.v. injection the mice were killed and their organs, blood and tumours harvested for counting. Tumour localisation was four times greater than that into normal tissues, reaching 20% injected dose per gram of tumour.

Eight patients have had this Tc99m-ICR12. Patient selection was by immunocytochemical staining of fine needle aspirates from the patient's own breast cancer. After intravenous administration of the immunoconjugate, tomographic images were obtained at 24 h. These results were compared to the subsequent histopathological examinations. Three patients acted as normal controls, one patient was negative due to inappropriate sampling, and two patients had strong membrane staining and provided excellent tumour localisation to both breast primary and regional node metastases. A further two patients only had moderate antigen expression on staining and did not localise well.

The good performance of this radiolabelled antibody with patients that strongly stain for the antigen encourages the development of this system as both a method of staging breast cancer and a potential means of immunotherapy in this subgroup of patients.

Radioimmunolocalisation, the use of radiolabelled monoclonal antibodies to localise tumour deposits displaying the target antigen, has been used in the staging of breast cancer with varied results (Tjandra *et al.*, 1989*a*,*b*; Athanassiou *et al.*, 1988). Successful localisation of an antibody to the target antigen relies firstly on there being a high level of antigen expression by the tumour relative to normal tissues (Weinstein *et al.*, 1984; Vaickus & Foon, 1991) and secondly, the availability of highly specific antibodies which recognise the target antigen. This tumour specificity is itself an essential prerequisite to any future development of immunotherapeutic applications (Bradwell *et al.*, 1985; Schlom, 1989). Most previously reported studies have used a single monoclonal antibody of moderate specificity for a tumour to investigate the potential for staging all cases of that cancer type under investigation (Thompson *et al.*, 1984; Yiu *et al.*, 1991), without making allowance for heterogeneity of antigen expression either between different tumour deposits or within a tumour itself. In addition, the level of antigen expression in many tumours is not signficantly elevated above that found on normal tissues, thus making successful tumour localisation unlikely.

We have tried to circumvent these problems of variable antigen expression by using a panel of monoclonal antibodies that are highly specific to clearly defined, but not universally expressed, cell surface antigens. By selecting the antibody for radioimmunolocalisation studies on the basis of prior immunostaining of fine needle aspirates of each tumour we aimed to identify the best antibody to be used for the investigation of each individual patient. We present the results from the first of our antibodies, ICR12 (Styles *et al.*, 1990) that has been fully evaluated both *in vitro*, *in vivo* and in clinical cases. This antibody, which is a rat IgG2a monoclonal, is directed against the extracellular domain of the 185 kDa product of the c-*erb*B-2 proto-oncogene; a transmembrane tyrosine kinase related to the receptor for epidermal growth factor. Amplification of this gene and overexpression of the product has been found in some 20% of breast cancers and overexpression has been found to correlate with poor prognosis in these patients (Slamon *et al.*, 1987; Gusterson *et al.*, 1988). The minimal levels of expression of this antigen in most normal tissues make this a highly tumour specific target (Perren, 1992). ICR12 was selected from a panel of rat monoclonal antibodies to p185 (Dean *et al.*, 1992) on the basis that it binds both to frozen and formalin fixed paraffin embedded tumour sections that overexpress p185 and it will localise with high efficiency and stability to p185 expressing breast and ovarian xenografts in athymic mice (Bakir *et al.*, 1992).

Correspondence: S.M. Allan, Academic Department of Surgery, Royal Marsden Hospital, Fulham Road, London SW3 6JJ, UK.
Received 29 September 1992; and in revised form 17 November 1992.

### Methods

### *Patient selection*

Patients were recruited for inclusion into this study from staging clinics at The Royal Marsden Hospital, Sutton. All patients had primary breast cancer, were aged between 35–80 years and were recruited prior to surgical management of the axilla. Written consent was obtained from each patient following approval of the protocol by The Royal Marsden Hospital Ethical Committee. The diagnosis of breast cancer relied on the triple approach of clinical findings, mammography and positive fine needle aspiration cytopathology or core biopsy histopathology.

### *Immunostaining*

Tumour cells obtained by fine needle aspiration were suspended in minimum essential medium with 25 mMolar HEPES buffer and phenol red. Aliquots of the suspension were cytocentrifuged using the Shandon cytospin centrifuge to provide an adequate number of equivalent samples for both cytopathology and immunocytochemical staining (Fernando *et al.*, 1992*a*). Immunostaining was by the indirect imunoperoxidase technique (Fernando *et al.*, 1992*b*) (Figure 1) and the results obtained with the cytospin samples were verified by staining of fixed paraffin embedded tissue obtained from the surgically resected specimen (Fernando *et al.*, 1992*b*). Strong staining was defined as dense immunoperoxidase brown staining of greater than 50% of malignant cell membranes. Whereas, moderate staining was defined by us as some evidence of brown staining being present on less than 50% of malignant cell membranes within any one high power microscopic field of view.

*Radiolabelling procedure*

After consideration of previous studies with differing isotopes and differing labelling methods including chelation methods of labelling with Tc99m, Technetium-99m was selected as the radioisotope for use with a reduction method of labelling because of the short half life, excellent imaging characteristics and ease of radiolabelling. ICR12 is labelled with Tc99m using a modification of the Mercaptoethanol reduction method (Schwarz & Steinstraesser, 1987; Mather & Ellison, 1990). Initial tests showed that the optimal labelling conditions for ICR12 with minimal affect on antibody binding required a modification of the standard technique. Briefly, the optimal conditions were achieved by treatment of the antibody by part reduction with 2-mercaptoethanol (2ME) at a molar ratio of 500 parts 2ME:1 part antibody, for 30 min at room temperature. The partially reduced antibody was separated from excess reductant by passage through a 10 by 1 cm column of Sephadex G25 previously equilibrated with phosphate buffered saline (pH 7.4) and blocked with human serum albumin. The partially reduced antibody elution fraction was both identified and quantified by measuring the optical density of the fractions at 280 nm wavelength against a saline control. The identified protein containing fraction is then frozen at − 20°C prior to completion of the labelling process. The final stage of labelling involves the further reduction of the partly reduced antibody using a standard bone scanning kit 'Osteoscan HDP' (Mallinckrodt Medical) at 100 microlitres from the reconstituted kit, (stannous chloride and disodium oxidronate, one vial reconstituted with 5 ml phosphate buffered saline) per milligram of the reduced antibody in the presence of 1000 MBq of Tc99m. Further gel filtration removed any non-protein bound Tc99m and the final labelled antibody preparation was then sterilised by microfiltration using a 0.22 micron low protein loss membrane filter (Millex-GV, Millipore). This technique of preparation has been tested for sterility and pyrogenicity by accredited commercial quality control testing laboratories. Labelling efficiency was checked prior to use of each batch of

**Figure 1** Membrane staining showing positive immunostaining on cytospin preparation from fine needle aspirate of breast primary.

prepared antibody by instant thin layer chromatography.

In the preclinical studies, the effects of differing conditions of radiolabelling on the immunoreactivity of the antibody were established using competitive radioimmunoassay techniques (Bakir *et al.*, 1992). ICR12 labelled with Tc99m (decayed) was compared with unlabelled ICR12 for its capacity to compete with Iodine-125 labelled ICR12 for binding to the human ovarian carcinoma cell line SKOV3 cells which over expresses the target antigen (Bakir *et al.*, 1992) (Figure 2). Standard competitive radioimmunoassay techniques were employed with doubling dilutions of each test antibody against the I-125-antibody down the cell plate. Further determination of the effect of labelling on immunoreactivity of the radiolabelled antibody was performed by immunoprecipitation studies of Tc99m labelled antibody binding to Sepharose 4B beads coated with an excess of the p185 antigen. The resultant trapping of the active, labelled antibody to the antigen coated beads was measured in an automated gamma counter. This method of testing demonstrates the combined effects of both labelling efficiency and immunoreactivity, rather than testing each function in isolation.

### *Biodistribution studies* in vivo

Eight athymic mice bearing established bilateral xenografts of the human breast carcinoma MDA MB-361 tumours were injected intravenously with 100 micrograms of Tc99m-ICR12 and then killed at 24 h by $CO_2$ asphyxiation. Radioactivity in weighed samples of blood, lungs, liver, spleen, kidneys, muscle, skin and tumours was determined in a gamma well spectrometer and the results expressed in terms of per cent injected dose per gram of tissue (% ID $g^{-1}$).

### *Imaging technique*

Patients were given 2 mg of ICR12 labelled with 700 MBq of Tc99m by slow intravenous injection. Axillary and thoracic regions were imaged at 24 h after injection using a General Electric Maxi gamma camera with a standard collimator. Planar acquisition of approximately 200,000 counts was performed initially, followed by 360 degree tomography acquiring in 64 projections for 30 s at each angle. Computerised reconstruction was then undertaken to provide tomographic representations in the transaxial, sagittal and coronal planes. Reconstruction of images was performed using a ramp filter, and these transaxial reconstructions were analysed further by computer to show levels of activity in regions of interest thus quantifying the imaged results.

**Figure 2** Competitive radioimmunoassay of unlabelled ICR12 (control) *vs* Tc99m labelled ICR12 (decayed) on Skov 3 cell plates against I-125 labelled ICR12.

### Results

### *Radiolabelling of ICR12 with Tc99m*

Experimentation was carried out initially to determine the optimum conditions for reduction of the antibody with 2ME. The conditions were varied in each experiment in terms of the molar ratios and the results of these experiments compared in terms of labelling efficiency, immunoreactivity and structural damage to the antibody. The process of labelling was demonstrated not to damage the structural integrity of the antibody by SDS-PAGE gel electrophoresis studies and the function of the antibody was demonstrated to be unaffected by the process of radiolabelling by competitive radioimmunoassay against Iodine-125 labelled ICR12 on SKOV 3 cell plates. The results of these experiments demonstrated the optimum labelling conditions for ICR12 and these differed for this rat monoclonal antibody from the originally reported technique describing murine antibodies. The modified protocol produced satisfactory labelling with Tc99m, such that the labelled antibody was indistinguishable from the Iodine labelled control antibody in terms of immunoreactivity. Labelling efficiency was checked on each preparation by instant thin layer chromatography using normal saline as the solvent carrier and these results were confirmed in the initial instances by high pressure liquid chromatography. Labelling efficiency had to exceed 95% (after purification by gel filtration) for satisfactory labelling to be accepted and this level of efficiency was demonstrated to be stable for a duration of 24 h *in vitro* and in animal models. Combined immunoreactivity and labelling efficiency was assessed by competitive immunoprecipitation assay on antigen linked Sepharose 4B beads and shown to be in the region of the potential maximum value, which compared favourably to those results obtained with 124-Iodine labelled ICR12 (Bakir *et al.*, 1992). This combined test showed that whilst maintaining very high labelling efficiency levels, the reduction method of labelling did not produce loss of immunoreactivity when compared to more established labelling methods.

### *Biodistribution of Tc99m-ICR12 in athymic mice bearing MDA MB-361 xenografts*

To determine the effect of the radiolabelling procedure on the biodistribution and tumour localisation of ICR12 studies were undertaken within a nude mouse model system. Eight athymic mice bearing bilateral subcutaneous human xenograft tumours of MDA MB-361 were injected intravenously with 100 micrograms of Tc99m labelled ICR12 and killed at 24 h. Imaging on a gamma camera was performed at this point (Figure 3) and various organs and tissues harvested for counting in an automated gamma well counter. The results determined as percentage injected dose per gram of tissue for the various tissues samples at 24 h are shown in Figure 4. The blood and normal tissue activity levels were comparable to those found in a similar study using Iodine-124 with the exception of the level of kidney uptake which may well represent the renal excretion of the radiolabel, or a small amount of free Tc99m present in the preparation. After 24 h Tc99m-ICR12 localised into tumour on average four times greater than any other normal tissue. Blood levels were half those in the tumour at 24 h and the mean tumour localisation was 20.7% of the total injected dose per gram weight of tumour (range 13.8% to 32.4% ID $g^{-1}$). These results of tumour localisation were the highest mean value obtained with this xenograft test system using ICR12 labelled with several different radionuclides and labelling methods including Tc99m wlth a chelation labelling technique (Dean *et al.*, 1992). We concluded that not only were the biological and immunological properties of ICR12 not detectably altered by this method of labelling with Tc99m, but that this is an excellent method of producing radiolabelled antibodies for clinical use. To ensure that the antibody preparations for clinical studies remained at such high quality, samples of the

**Figure 3** Image of nu/nu mouse bearing bilateral flank tumours (MDA MB 361 xenografts) taken at 24 h post injection.

**Figure 4** Mean percentage injected dose Tc99m-ICR12 per gram of tissue at 24 h in mouse model system.

clinical investigation preparations were tested in this animal model in tandem to clinical studies. This allowed confirmation of the labelled antibody immunoreactivity in each clinical trial case, thus acting as a positive control.

*Patient studies*

To date eight patients have been studied with Tc99m-ICR12, (Table I) three of these acted as normal controls since their tumours did not show over expression of the gene product on their cell membranes in either their fine needle aspirates or their surgical resection specimen paraffin embedded sections from the tumour or axillary lymph nodes.

Two patients had marked over expression of the antigen on pretesting. In the first case (Patient 4) excellent images were obtained on tomographic reconstruction of both the primary and nodal tumour deposits. The second case (Patient 5) had even stronger immunostaining on pretesting with

Table I Clinical results with Tc99M-ICR12

| Patient | Clinical node status | Antigen expression | Histological node status | Immuno-localisation result |
|---|---|---|---|---|
| 1 | – | – | + | – |
| 2 | + | – | + | – |
| 3 | – | + (Only in DCIS) | – | – |
| 4 | + | + + + | + | + + |
| 5 | + | + + + | + | + + |
| 6 | + | – | + | – |
| 7 | – | + (Moderate) | – | +/– |
| 8 | + | + (Moderate) | + | +/– |

DCIS = Ductal carcinoma *in situ*; Moderate = <50% cells showing some membrane immunostaining.

marked homogeneity of the neoplastic cell staining. Radioimmunolocalisation images easily identified the tumour deposits on both planar imaging (Figure 5) as well as the computer enhanced tomographic reconstructions (Figure 6). The reconstructed transaxials were then further examined to show region of interest axes and the highlighted tumour deposits showed levels of activity at least three times that of background admixture of blood and normal tissue levels of activity (Figure 7). The line axes show activity levels through both breasts along the horizontal axis, while the vertical axis compares the total body mass and blood background against the tumour activity level.

One patient selected for inclusion in the study as a 'positive expresser' of the c-*erb*B-2 p185 on the basis of staining of the fine needle aspirate failed to localise on radioimmunoscintigraphy with Tc99m-ICR12 (Patient 3). Immunostaining of the surgically resected specimens showed that whilst there was over expression of the gene product on surrounding ductal cancer *in situ*, the infiltrative component of the tumour which formed the bulk of the tumour mass did not stain. The tandem mouse study showed that the labelled antibody behaved as expected. We concluded that this case represented a problem of sampling by fine needle aspiration rather than a problem with the monoclonal antibody.

Two further patients had only weak to moderate expression of the antigen on their breast cancer cell membranes as shown by pretesting immunostaining. In addition both of these patients had had three or more months of presurgical treatment with Tamoxifen as part of a primary medical treatment programme. Neither of these two patients demonstrated good localisation to either the breast primary or the axillary nodes with Tc99m-ICR12 radioimmunolocalisation. Whether this was due to the lower degree of antigen expression being displayed by the target tumour, or whether the previous hormonal treatment had in some way modified the immunological target remains to be clarified.

### Discussion

Our results from the preclinical evaluation experiments and the subsequent clinical evaluation provide support for the hypothesis that the use of a highly specific antibody against a preselected target will improve the prospects for accurate localisation of tumour deposits by radioimmunolocalisation. The assessment of the efficiency of staining of fine needle aspirates for characterisation of the tumour target has given good agreement with definitive pathological examination and immunocytochemical (Fernando *et al.*, 1992*b*) although one result (Patient 3) points to the need for careful sampling.

The results of this radioimmunolocalisation study support our hypothesis that in order to improve on previously reported results where a single monoclonal antibody is expected to localise in all cases of a particular tumour type. We recognised that this is asking too much of any individual antibody and have therefore selected antibodies to form a panel from which an antibody is targeted to a specific and predetermined target antigen. By having a panel of highly specific antibodies, from which one is chosen on the basis of testing each individual tumour target antigen expression, the ability of the chosen antibody to recognise that particular

**Figure 5** Planar image from patient five, showing both primary and node uptake.

**Figure 6** Transaxial, coronal and sagittal reconstruction slices through the axilla, showing uptake in the node metastases in patient five.

**Figure 7** Transaxial reconstruction showing levels of activity along axes lines of interest through the breast primary tumour of patient five.

individual tumour being tested is therefore established prior to the study. It is in using this method of preselecting antibodies for the task in hand that radioimmunolocalisation will be more likely to provide accurate data for staging purposes. We do however recognise that the extent of the antigen expression and any modifying factors such as pre-treatment should be taken into consideration in this selection process.

The results presented in this paper are the initial results of the first antibody from our panel of highly specific antibodies, and show the potential of such a directed means of investigation. The excellent localising properties of ICR12 into tumours that strongly over express c-*erb*B-2 p 185 and the absence of uptake of this antibody in antigen negative tumours and normal breast tissue suggest that it may have a useful clinical role in staging patients and should be included as a constituent member of our eventual completed panel of antibodies for staging breast cancer.

Recent reports (Wright *et al.*, 1992; Gusterson *et al.*, 1992) suggest that breast cancer patients with tumours that over express the product of the c-*erb*B-2 proto-oncogenes have a poor prognosis because the tumours are more resistant to chemotherapy or hormonal manipulation. The results of our initial study with ICR12 reported in this paper suggest that the good tumour localising properties of this antibody in those patients that strongly overexpress the target antigen may form a basis for its use in therapeutic applications in these patients, for example in antibody directed prodrug therapy or targeted immunotherapy with either antibody targeted toxins or antibody delivered radiotherapy. In conclusion, not only could ICR12 form part of a panel of antibodies to stage breast cancer and thereby replace axillary surgery as a diagnostic procedure in this condition, but also that the antibody may be of use for delivering and directing subsequent therapeutic management in those patients that strongly over express the c-*erb*B-2 gene product.

We thank Mr A.G. Nash and Dr T.J. Powles for the permission to use their patients in this study.
We thank Miss J. Sandle for technical assistance in immunostaining.

### References

ATHANASSIOU, A., PECTASIDES, D., PATENIOTIS, K., TZIMIS, L., NATSIS, P., LAFI, A., ARAPANTONI, P., KOUTSIOUBA, P., TAYLOR-PAPADMIMITRIOU, J. & EPENETOS, A. (1988). Immunoscintigraphy with 131 I labelled HMGF2 and HMFG1 F(AB')2 in the pre-operative detection of clinical and subclinical lymph node metastases in breast cancer patients. *Int. J. Cancer*, (Suppl.) **3**, 89-95.

BAKIR, M.A., ECCLES, S.A., BABICH, J.W., AFTAB, N., STYLES, J.M., DEAN, C.J. & OTT, R.J. (1992). C-erbB-2 protein overexpression in breast cancer as a target for P.E.T. using 124-I labelled monoclonal antibodies. *J. Nucl. Med.*, **33**, 2154-2160.

BRADWELL, A.R., FAIRWEATHER, D.S., DYKES, P.W., KEELING, A., VAUGHAN, A. & TAYLOR, J. (1985). Limiting factors in the localisation of tumours with radiolabelled antibodies. *Immunol. Today*, **6**, 163-170.

DEAN, C.J., STYLES, J., VALERI, M., MODJTAHEDI, H., BAKIR, A., BABICH, J.W. & ECCLES, S. (1992). *Growth Factor Receptors as Targets for Antibody Therapy Mutant Oncogenes: Targets for Therapy?* Epenetos, A.A. & Lemoine, N. (eds) 27-34.

FERNANDO, I.N., MCROBERT, L., SMITH, D., TROTT, P.A., DOWSETT, M. & POWLES, T.J. (1992*a*). Determination of immunocytochemical prognostic factors in breast cancer using a single fine needle aspirate. *Br. J. Cancer*, (Suppl.) **66**, 8, 030.

FERNANDO, I.N., ALLAN, S.M., SANDLE, J., SACKS, N.P.M. & TROTT, P.A. (1992*b*). The role of fine needle aspiration cytology and the cytospin technique in determining immunocytochemical staining for the C-erbB-2 gene product. *Cytopathology*, (in press).

GUSTERSON, B.E. *et al.* FOR THE INTERNATIONAL (LUDWIG) BREAST CANCER STUDY GROUP (1992). Prognostic importance of C-erbB-2 expression in breast cancer. *J. Clin. Oncol.*, **10**, 1049- 1056.

GUSTERSON, B.A., MACHIN, L.G., GULLICK, W.J., GIBBS, N.M., POWLES, T.J., PRICE, P., MCKINNA, A. & HARRISON, S. (1988). Immunohistochemical distribution of C-erbB-2 in infiltrating and *in situ* breast cancer. *Int. J. Cancer*, **42**, 842-845.

MATHER, S.J. & ELLISON, D. (1990). Reduction-mediated technetium-99M labelling of monoclonal antibodies. *J. Nucl. Med.*, **31**, 692-697.

PERREN, T.J. (1991). CerbB2 oncogene as a prognostic marker in breast cancer. *Br. J. Cancer*, **63**, 328-332.

SCHLOM, J. (1989). Innovations in monoclonal antibody tumour targeting. *J. Amer. Med. Assoc.*, **261**, 744-746.

SCHWARZ, A. & STEINSTRAESSER, A. (1987). A novel approach to Tc-99m-labelled monoclonal antibodies. *J. Nucl. Med.*, **28**, 721: A695.

SLAMON, D.J., CLARK, G.M., WONG, S.G., LEVIN, W.J., ULLRICH, A. & MCGUIRE, W.L. (1987). Human breast cancer: correlation of relapse and survival with amplification of the Her-2/Neu oncogene. *Science*, **235**, 177-182.

STYLES, J.M., HARRISON, S., GUSTERSON, B.A. & DEAN, C.J. (1990). Rat monoclonal antibodies to the external domain of the product of the C-erbB-2 proto-oncogene. *Int. J. Cancer*, **45**, 320-324.

THOMPSON, C.H., STACKER, S.A., SALEHI, N., LICHTENSTEIN, M., LEYDEN, M., ANDREWS, J.T. & MCKENZIE, I.F.C. (1984). Immunoscintigraphy for the detection of lymph node metastases from breast cancer. *Lancet*, **2**, 1245-1247.

TJANDRA, J.J., RUSSELL, I.S., COLLINS, J.P., ANDREWS, J.T., LICHTENSTEIN, M., BINNS, D. & MCKENZIE, I.F.C. (1989*a*). Immunolymphoscintigraphy for the detection of lymph node metastases from breast cancer. *Cancer Res.*, **49**, 1600-1608.

TJANDRA, J.J., SACKS, N.P.M., THOMPSON, C.H., LEYDEN, M.J., STACKER, S.A., LICHTENSTEIN, M., RUSSELL, I.S., COLLINS, J.P., ANDREWS, J.T., PIETERSZ, G.A. & MCKENZIE, I.F.C. (1989*b*). The detection of axillary lymph node metastases from breast cancer by radiolabelled monoclonal antibodies: a prospective study. *Br. J. Cancer*, **59**, 296-302.

VAICKUS, L. & FOON, K.A. (1991). Overview of monoclonal antibodies in the diagnosis and therapy of cancer. *Cancer Invest.*, **9**, 195-209.

WEINSTEIN, J.N., COVELL, D.G., KEENAN, A.M., HOLTON, O.D., STELLER, M.A., SIEBER, S.M., LARSON, S.M., SPAULDING, G.F. & PARKER, R.J. (1984). Optimisation of immunolymphoscintigraphy. *Hybridoma*, **3**, 66.

WRIGHT, C., NICHOLSON, S., ANGUS, B., SAINSBURY, J.R.C., FARNDON, J., CAIRNS, J., HARRIS, A.L. & HORNE, C.H.W. (1992). Relationship between c-erbB2- protein product expression and response to endocrine therapy in advanced breast cancer. *Br. J. Cancer*, **65**, 118-121.

YIU, C.Y., BAKER, L.A. & BOULOS, P.B. (1991). Anti-epithelial membrane antigen monoclonal antibodies and radioimmunolocalisation of colorectal cancer. *Br. J. Surg.*, **78**, 1212-1215.